# Comparison of the Effect of Low-Frequency Repetitive Transcranial Magnetic Stimulation with That of Theta Burst Stimulation on Upper Limb Motor Function in Poststroke Patients

**DOI:** 10.1155/2017/4269435

**Published:** 2017-11-05

**Authors:** Takahiro Kondo, Naoki Yamada, Ryo Momosaki, Masato Shimizu, Masahiro Abo

**Affiliations:** ^1^Shimizu Hospital, Kurayoshi-City, Tottori, Japan; ^2^Department of Rehabilitation Medicine, The Jikei University School of Medicine, Tokyo, Japan

## Abstract

**Background:**

The purpose of this study was to evaluate the difference between the therapeutic effect of low-frequency repetitive transcranial magnetic stimulation (LF-rTMS) and that of continuous theta burst stimulation (cTBS), when each is combined with intensive occupational therapy (OT), in poststroke patients with upper limb hemiparesis.

**Materials and Methods:**

The study subjects were 103 poststroke patients with upper limb hemiparesis, who were divided into two groups: the LF-rTMS group (*n* = 71) and the cTBS group (three pulse bursts at 50 Hz) (*n* = 32). Each subject received 12 sessions of repetitive transcranial magnetic stimulation of 2,400 pulses applied to the nonlesional hemisphere and 240-min intensive OT (two 60-min one-to-one training sessions and two 60-min self-training exercises) daily for 15 days. Motor function was evaluated using the Fugl-Meyer Assessment (FMA) and the performance time of the Wolf motor function test (WMFT) was determined on the days of admission and discharge.

**Results:**

Both groups showed a significant increase in the FMA score and a short log performance time of the WMFT (*p* < 0.001), but the increase in the FMA score was higher in the LF-rTMS group than the cTBS group (*p* < 0.05).

**Conclusion:**

We recommend the use of 2400 pulses of LF-rTMS/OT for 2 weeks as treatment for hemiparetic patients.

## 1. Introduction

The application of transcranial magnetic stimulation (TMS) to the cerebral cortex was first described in 1985 by Barker et al. [1], who demonstrated that such stimulation increased the conduction of nerve impulses from the motor cortex to the spinal cord, and resulted in hand muscle contractions. More recently, TMS has been used as a therapeutic modality for various diseases [2–5]. Stimulation by TMS has been applied using variations in parameters, such as frequency of TMS, stimulation space, stimulation intensity, stimulation site, and duration of stimulation time. Repetitive TMS (rTMS) is a noninvasive method used for the stimulation of selected brain areas and has been demonstrated to modulate cortical excitability and function depending on the frequency of stimulation. Low-frequency rTMS (LF-rTMS) of ≤1 Hz suppresses local neural activities, while high-frequency rTMS (HF-rTMS) of ≥5 Hz activates local neural activities [6, 7]. In recent years, continuous theta burst stimulation (cTBS) and intermittent theta burst stimulation (iTBS) have been described as two forms of rTMS [8, 9]. The TBS protocol comprises bursts of three pulses at 50 Hz. The stimulation pattern is either excitatory (iTBS) or inhibitory (cTBS) on brain activity [8]. TBS can control the activation of local nerve activity through very short duration stimulation.

Randomized controlled trials have shown that the application of LF-rTMS to the nonlesional hemisphere significantly improves motor function of the affected upper limb in poststroke hemiparetic patients, through indirect activation of the lesional hemisphere [4, 10–12].

Although not a randomized controlled trial, our group has reported that both TMS patterns (LF-rTMS and cTBS) combined with occupational therapy (OT) can improve poststroke motor function of the hemiparetic upper limb [13, 14]. However, in that study, because of the small target group, the stimulation number could not be verified as optimal. In addition, cTBS or LF-rTMS could not be compared in terms of the recovery effect for functional arm movements of patients recovering after brain damage stroke. Various TMS stimulation methods are available; however, our clinic was not able to determine the most effective method for each patient. The purpose of this study was to compare the effects of LF-rTMS and cTBS on functional arm movements in poststroke patients with motor paralysis due to brain damage in order to determine the most effective method.

## 2. Subjects and Methods

All patients were referred to Shimizu Hospital or the Department of Rehabilitation Medicine, The Jikei University School of Medicine, for suitability of in-patient treatment with rTMS. The study was approved by the local ethics review committee of our hospital and informed consent was obtained from each patient before the study.

The subjects were 103 poststroke patients with spastic upper limb hemiparesis that were admitted to our hospital from February 20, 2010, to April 4, 2015. Seventy-one patients admitted before February 2, 2013, received LF-rTMS/OT, whereas 32 patients admitted after February 2, 2013, received cTBS/OT. Inclusion criteria were based on the TMS guidelines of Wassermann et al. [13, 15] and included the following: (1) upper limb hemiparesis categorized as cerebral infarction or cerebral hemorrhage, (2) age between 18 and 70 years, (3) minimal time since stroke of 12 months, (4) history of a single stroke only (no bilateral cerebrovascular lesions), (5) no cognitive deficits (a Mini Mental State Examination score of ≥26), (6) no active physical or mental illness requiring medical management, (7) no history of convulsions for at least one year, (8) no intracranial metal clips or intracardiac pacemaker, (9) no history of neurolytic nerve block (phenol or botulinum toxin) to the affected upper limb, and (10) severity of upper limb hemiparesis of Brunnstrom recovery stages 3 to 5 for hand-fingers muscles.  Table 1 summarizes the clinical characteristics of the patients. The mean age of all the patients was 61.5 ± 13.0 years (±SD). The time between stroke onset and treatment ranged from 12 to 270 months with a mean of 68.0 ± 57.5 months. Stroke was classified as intracerebral hemorrhage in 41 patients (39.8%) and cerebral infarction in 62 patients (60.2%). There was no significant difference in the clinical parameters between groups except in the two types of brain damage.

All patients were hospitalized for 15 days to receive LF-rTMS/OT or cTBS/OT. During hospitalization, each patient received one 40-min session of LF-rTMS or 160-sec session of cTBS, plus two sessions of intensive OT daily, except for the days of admission/discharge and Sundays (Table 2). In the following statistical processing, in addition to the comparison of LF-rTMS and cTBS, LF-rTMS and TBS were also compared according to the significant difference between groups in the type of brain damage.

### 2.1. Application of LF-rTMS (2400 Pulses) and cTBS (2400 Pulses)

A 70-mm figure-8 coil attached to MagPro R30 stimulator (MagVenture Company, Farum, Denmark) was used for application of rTMS. For LF-rTMS, 2,400 pulses lasting 40 min were applied per session. The intensity of stimulation was set at 90% of resting motor threshold of the first dorsal interosseous (FDI) muscle, which was defined as the lowest intensity of stimulation that could activate motor evoked potentials (MEPs) of the FDI muscle. One session of long-duration cTBS protocol comprised application of bursts of three pulses at 50 Hz, repeated every 200 ms intervals (i.e., at 5 Hz). The total duration of stimulation was 160-sec (total; 2400 pulses). The intensity of stimulation was set at 80% of the motor threshold of the FDI muscle. All patients were monitored carefully by the physician during the application of rTMS.

### 2.2. Occupational Therapy and Unsupervised Training

OT involved one-to-one individual 60-min training sessions performed twice a day, 6 days a week (excluding Sundays) [13]. Unsupervised training was performed in a quiet place to avoid interference such as verbal or visual interference by other patients. Upon completion of the unsupervised training, the occupational therapist checked each patient's task performance through an interview and helped the patient reflect on the next independent training session (such as with the addition of new tasks). In the interview at the time of the voluntary training, the degree of difficulty was estimated in terms of training accomplishments and feelings of being tired; plus training sessions were conducted by combining occupational therapy conditions with patient individuality.

The main goal of the OT and unsupervised training was to help the patients avoid focusing mainly on functional training, to allow the patients to use their affected upper limb again in daily situations, and to encourage patients to use the paralyzed upper limb in daily situations. The treatment strategy included (1) incorporation of a fair amount of everyday physical activity in the training tasks, (2) individualized functional training serving to acquire some movements and activities, (3) incorporation of elements involved in gross motor function, fine motor function, and multitasking, (4) clear demonstration of the position of the upper limb to draw attention of the like during training, (5) enabling specific staged intervention, (6) incorporation of content that can be continued at home after discharge in situations involving activities of daily life (ADL) and unsupervised training, (7) not restraining the paralyzed upper limb such as in constraint-induced movement therapy (CIMT), and (8) the provision of action feedback by passive intervention with verbal instructions.

Repetitive training included various tasks such as wiping the table with a dust cloth, picking up an object by coordinating the function and shape of the hand, and grasping and moving an object with chopsticks. These tasks were performed as active movements or active-assisted movements. Details of the training varied according to the severity of poststroke disability, lifestyle, and goals, and rehabilitation was performed as required at the time.

### 2.3. Clinical Evaluation of Motor Function

Motor function of the affected upper limb was evaluated on the days of admission and discharge by Fugl-Meyer Assessment (FMA) and Wolf Motor Function Test (WMFT). The FMA was devised in 1975 by Fugl-Meyer et al. [16] and is a global assessment index used to quantitatively evaluate the recovery of poststroke hemiparetic limbs. Both the FMA and WMFT have high interrater and test-retest reliability, as described previously [17, 18]. The FMA (a performance-based quantitative measure) comprises 33 items that evaluate upper limb motor function. Since each item is rated on a three-point ordinal scale (0 = cannot perform, 1 = can perform partially, and 2 = can perform fully), 66 points is the maximal score for motor performance of the upper limb. The WMFT comprises 15 timed tasks (6 exercise tasks and 9 article operation tasks) that evaluate upper limb motor function. The mean performance time of the 15 tasks was calculated [19]. When the task was not completed within 120 sec, the task performance time was recorded as 120 seconds. The WMFT performance time data showed a skewed distribution pattern and, thus, the data were converted to the natural logarithm before analysis, as described previously in the EXCITE trial [20].

### 2.4. Statistical Analysis

Changes in the FMA score and the WMFT log performance time (WMFT-lpt) as a result of the treatment were examined using signed Wilcoxon's rank sum test, respectively. Mann–Whitney *U* test was used for comparison of each change level of the FMA score and the WMFT-lpt between the LF-rTMS and cTBS. In addition, signed Wilcoxon's rank sum test was used to compare the changes in the FMA score and the WMFT-lpt of LF-rTMS and cTBS between cerebral infarction and cerebral hemorrhage. The subjects were divided into two groups according to the stroke type and their data were compared statistically using Mann–Whitney *U* test. A* p* value less than 0.05 was considered statistically significant. All statistical analyses were performed using the Statistical Package for Social Sciences, v19.0 (SPSS Inc., Chicago, IL).

## 3. Results

**Figure 1:**
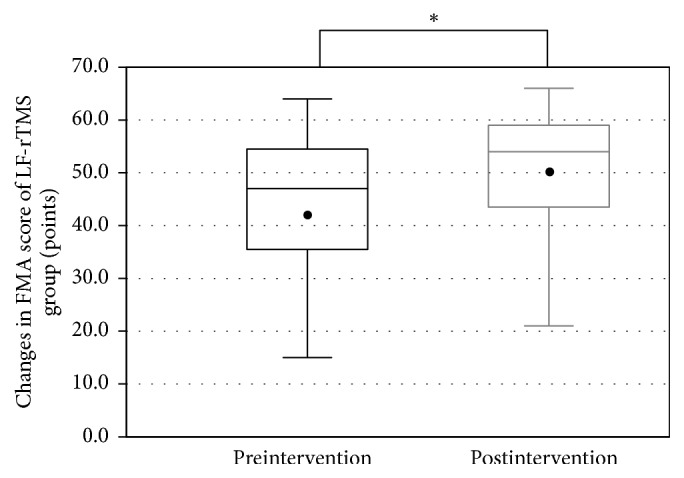
Changes in the FMA score of the TMS group with the intervention. A significant increase in the FMA score was observed (*p* < 0.001). ^*∗*^A statistically significant difference (*p* < .05).

**Figure 2:**
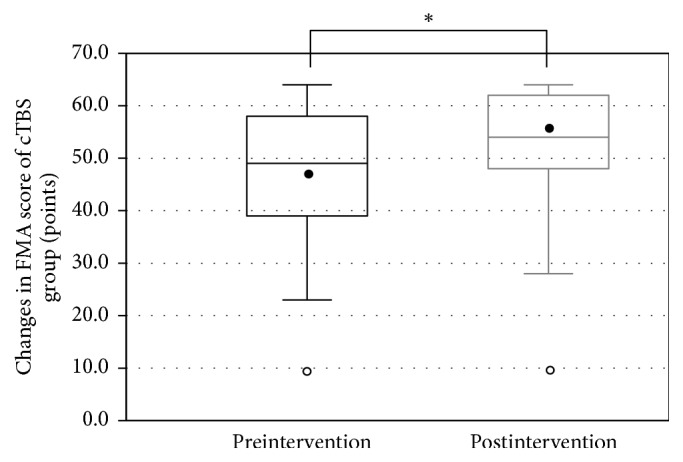
Changes in the FMA score of the TBS group with the intervention. A significant increase in the FMA score was observed (*p* < 0.001). ^*∗*^A statistically significant difference (*p* < .05).

**Figure 3:**
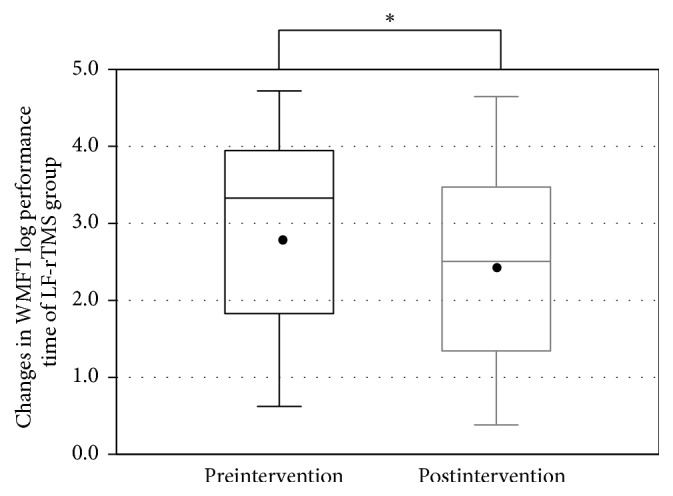
Changes in the WMFT log performance time of the TMS group with the intervention. A significant increase in the WMFT log performance time was observed (*p* < 0.001). ^*∗*^A statistically significant difference (*p* < .05).

**Figure 4:**
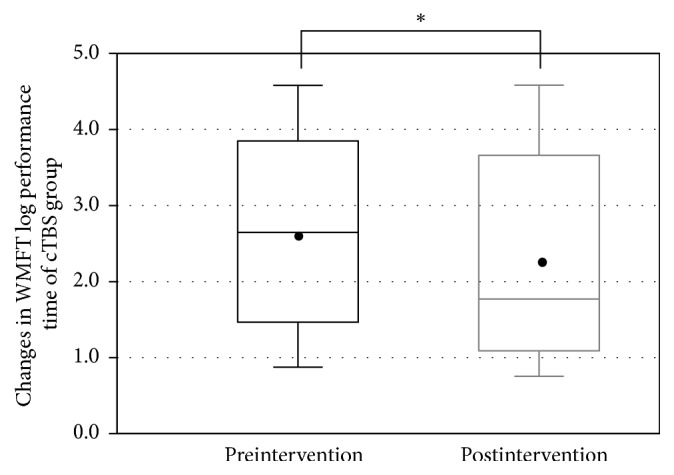
Changes in the WMFT log performance time of the TBS group with the intervention. A significant increase in the WMFT log performance time was observed (*p* < 0.001). ^*∗*^A statistically significant difference (*p* < .05).

**Figure 5:**
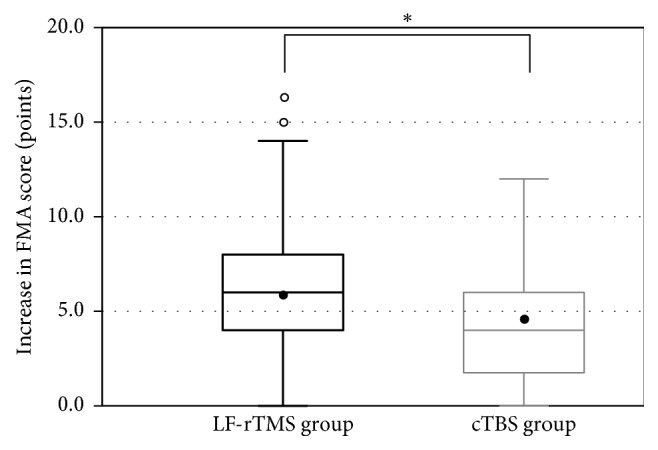
Increase in the FMA score with the intervention. The TMS group showed a significantly greater increase over that of the TBS group (*p* < 0.05). ^*∗*^A statistically significant difference (*p* < .05).

**Figure 6:**
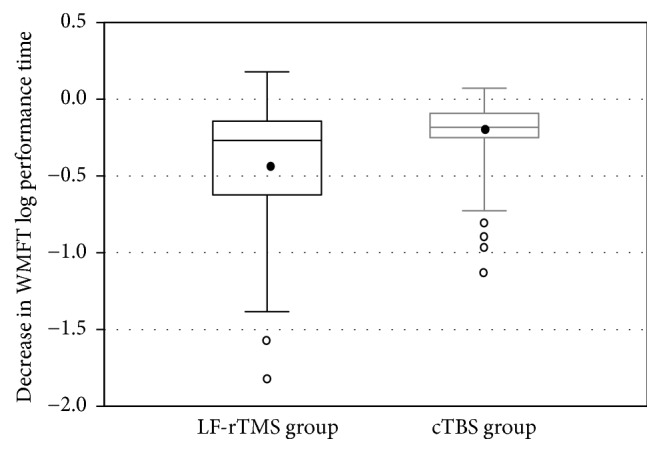
Decrease in the WMFT log performance time with the intervention. The decrease in the WMFT log performance time showed no significant difference between the TMS and TBS groups (*p* = 0.067).

All patients completed the LF-rTMS/OT and cTBS/OT protocols without any adverse effects. The FMA score was recorded successfully before and after treatment in both groups (LF-rTMS/OT: from 44.5 ± 12.3 to 50.5 ± 10.8 points, *p* < 0.001; cTBS/OT: from 47.1 ± 12.9 to 51.4 ± 12.2 points, *p* < 0.001) (Figures 1 and 2). Likewise, The WMFT-lpt decreased significantly in both groups (LF-rTMS/OT: from 2.93 ± 1.19 to 2.47 ± 1.22, *p* < 0.001; cTBS/OT: from 2.62 ± 1.22 to 2.33 ± 1.29, *p* < 0.001) (Figures 3 and 4). The mean increase in FMA score was 6.0 ± 3.5 points in LF-rTMS/OT group and 4.4 ± 3.3 points in cTBS/OT group. The increase was significantly larger in LF-rTMS/OT than cTBS/OT group (*p* < 0.05) (Figure 5). On the other hand, the mean decrease in WMFT log was 0.47 ± 0.47 in LF-rTMS/OT group and 0.29 ± 0.30 in cTBS/OT group. The difference in the mean decrease in WMFT-lpt was not significant between the two groups (*p* = 0.067) (Figure 6).

### 3.1. Results of Cerebral Infarction Patients

The FMA score was recorded before and after treatment in patients with cerebral infarction and was increased in both groups (LF-rTMS/OT: from 45.1 ± 11.5 to 51.1 ± 9.8 points, *p* < 0.001; cTBS/OT: from 47.4 ± 13.4 to 51.4 ± 13.0 points, *p* < 0.001). The mean increase in FMA score of LF-rTMS/OT group (6.1 ± 3.9 points) was significantly larger than that in cTBS/OT group (4.0 ± 3.3 points, *p* < 0.05). The WMFT-lpt of the same patient groups decreased significantly in both groups (LF-rTMS/OT: from 2.83 ± 1.22 to 2.38 ± 1.18, *p* < 0.001; cTBS/OT: from 2.42 ± 1.24 to 2.16 ± 1.29, *p* < 0.001), but the magnitude of the decrease in LF-rTMS/OT group (0.45 ± 0.44) exceeded that of cTBS/OT group (0.27 ± 0.29, *p* < 0.05).

### 3.2. Results of Cerebral Hemorrhage Patients

The FMA score was also recorded before and after the treatment in patients with cerebral hemorrhage. The treatment resulted in an increase in the score in both groups (LF-rTMS/OT: from 44.0 ± 13.2 to 49.8 ± 11.9 points, *p* < 0.001; cTBS/OT: from 45.7 ± 11.8 to 51.6 ± 9.6 points, *p* < 0.05). The mean increase in FMA score in patients with cerebral hemorrhage was not significantly different (LF-rTMS/OT: 5.9 ± 3.1 and cTBS/OT: 5.9 ± 3.6 points, *p* = 0.986). On the other hand, the WMFT-lpt decreased significantly in both groups (LF-rTMS/OT: from 3.05 ± 1.17 to 2.56 ± 1.28, *p* < 0.001; cTBS/OT: from 3.30 ± 0.93 to 2.93 ± 1.20, *p* < 0.05), and again, the mean decrease in WMFT-lpt was not significantly different between the two groups (LF-rTMS/OT: 0.49 ± 0.51 and cTBS/OT: 0.38 ± 0.36, *p* = 0.773).

## 4. Discussion

In the present study, although the number of pulses used in rTMS was similar in the two groups, the method of stimulation was different. Thus, we studied the difference in the effects of the two methods on upper limb motor function.

Previous studies indicated that LF-rTMS applied to the nonlesional cerebral hemisphere improves upper limb motor function in poststroke patients [12, 13, 21]. For example, Kakuda et al. [22] reported that the application of LF-rTMS to the nonlesional hemisphere combined with 120 min/day intensive OT for 15 days improved motor function and spasticity in 1700 poststroke patients. Furthermore, Avenanti et al. [23] reported that concurrent physiotherapy after application of LF-rTMS to the nonlesional hemisphere in poststroke hemiparetic patients reduced interhemispheric inhibition and readjusted hemispheric excitability, which improved motor function of the hemiparetic upper limb. In addition, Kondo et al. [24] reported that the combination therapy of LF-rTMS and OT in poststroke hemiparetic patients with spasticity reduced the F-wave parameter and improved spasticity. It is thought that LF-rTMS provides a state of imbalance in interhemispheric inhibition arising after stroke, improves plasticity of the affected hemisphere, and may help establish new neuronal circuits [4, 5, 25]. It is also thought that LF-rTMS promotes functional improvement in the central control of muscle output and may help regulate muscle tension. Therefore, we believe that a similar process was also operational in our patients, which resulted in posttreatment improvement.

With regard to TBS, Huang et al. [8] concluded that TBS (600 pulses) changed cortical excitability in humans. Furthermore, Yamada et al. [14] implemented a 15-day protocol of cTBS applied to the nonlesional hemisphere combined with intensive OT in poststroke patients. They concluded that the treatment was safe and improved motor function of the hemiparetic upper limbs. Considered together with our results, it seems that 2400-pulse cTBS effectively inhibits excitability of the nonlesional hemisphere, similar to LF-rTMS, and, in turn, promotes plasticity of the lesional hemisphere and improves motor function of the affected upper limb. In this regard, Gamboa et al. [26] compared 600-pulse cTBS with 1200-pulse cTBS and reported that while cTBS inhibited the stimulated hemisphere, it had an enhanced effect at 1200 pulses. In our study, cTBS was applied at a 4-fold stimulation rate compared with that used by Huang et al. [8], which could explain its enhanced effect. It is noteworthy that 2400-pulse cTBS may also have an inhibitory effect, and therefore further studies should be performed to determine the effects of 2400-pulse cTBS on the cerebral hemisphere. In another study, cTBS was applied 4 times per day using a 15-min interval between the first and the second sessions, 60 min between the second and third, and a 75-min interval between the third and fourth. They used 8 cTBS sessions over two days, in combination with 3 hrs of rehabilitation per day, and found ADL over a period of 3 weeks [27].

The main findings of the present study were improvement in motor function of the paralyzed upper limb in both the LF-rTMS and cTBS groups and a greater improvement in motor function of the upper limb after LF-rTMS/OT compared with cTBS/OT, especially in patients with cerebral infarction, though the effect was less significant in those with cerebral hemorrhage. Based on our results, we recommend the application of 2400-pulse LF-rTMS/OT for 2 weeks for hemiparetic patients with cerebral infarction.

Our results showed no difference in the improvement of motor function between the LF-rTMS and cTBS groups in patients with cerebral hemorrhage. While our study did not investigate the reason for the lack of difference, this finding is probably related to the relatively small number of subjects of this group. Further large-scale study is required to determine the effects of cTBS in poststroke patients with a history of cerebral hemorrhage.

TMS can be considered a tool to help the rehabilitation treatment to proceed more smoothly and effectively. However, it seems that the TMS protocol needs further fine-tuning to produce maximum effects in rehabilitation medicine. The effects of different stimulation methods of TMS in rehabilitation should be examined. When the applied TMS stimulation method has a facilitatory effect on the nonlesional hemisphere, rehabilitation should include more tasks that require movements of both hands rather than being limited to the paretic limb only. In this regard, the symptoms, clinical characteristics, environmental factors, and goals varied greatly among our stroke patients. Therefore, we believe that custom-tailored rehabilitation tasks should be considered for the individual patients.

The present study has certain limitations. First, we did not examine the immediate inhibitory effect at the time and immediately after the application of 2400-pulse cTBS to the nonlesional hemisphere using diagnostic tests and neuroimaging, such as fMRI, nerve conduction velocity, and neurophysiologic examination. Second, there was a large difference in the sample size of the two groups. Third, the long-term effects of the treatment were not examined after completion of the study. Fourth, the previous cTBS study was conducted using 600 pulses, and, though the interval was set due to increasing stimulation, we performed cTBS using 2400 pulses with no interval. Thus, it is necessary to conduct the same process for LF-rTMS stimulation.

By clarifying these issues, we can determine the best method associated with the best improvement in motor function of the paretic upper limb, before we can recommend the most effective therapy for poststroke hemiparetic patients.

## 5. Conclusion

Our proposed 15-day protocol of LF-rTMS combined with intensive OT may be a more useful therapeutic modality than cTBS/OT for upper limb hemiparesis after stroke. However, further studies are needed to confirm its efficacy.

## Figures and Tables

**Table 1:** Clinical characteristics of the 103 patients.

	TMS group (*n* = 71)	TBS group (*n* = 32)	*p* value
Age at admission, years^*∗*^	62.3 ± 12.5	60.0 ± 14.2	0.544
Time after onset of stroke, months^*∗*^	66.8 ± 53.9	70.6 ± 65.7	0.839
Female, *n* (%)	20 (28.2)	10 (31.3)	0.751
Subtype of stroke, *n* (%)			0.013
Intracerebral hemorrhage	34 (47.9)	7 (21.9)	
Cerebral infarction	37 (52.1)	25 (78.1)	
Side of hemiparesis, *n* (%)			0.866
Dominant hand	32 (45.1)	15 (46.9)	
Nondominant hand	39 (54.9)	17 (53.1)	
BRS for hand-fingers at admission, *n* (%)			0.889
Stage 3	12 (16.9)	5 (15.6)	
Stage 4	26 (36.6)	13 (40.6)	
Stage 5	33 (46.5)	14 (43.8)	
FMA, point^¶^	44.5 ± 12.3	47.1 ± 12.9	0.269
Sensory disturbance			0.272
Absent	50 (70.4)	19 (59.4)	
Present	21 (29.6)	13 (40.6)	

BRS: Brunnstrom recovery stage; FMA: Fugl-Mayer Assessment. ^*∗*^Data are mean ± SD. ^¶^Data are median (range).

**Table 2:** Protocol for LF-rTMS or cTBS and intensive occupational therapy.

	Saturday	Sunday	Monday–Friday	Saturday	Sunday	Monday–Friday	Saturday
Morning	Admission		LF-rTMS or cTBS (2400 pulses)		Rest day	LF-rTMS or cTBS (2400 pulses)	Posttherapy evaluation
		One-to-one training (60 min)	One-to-one training (60 min)	One-to-one training (60 min)		One-to-one training (60 min)	
		Self-exercise (60 min)	Self-exercise (60 min)	Self-exercise (60 min)		Self-exercise (60 min)	

Afternoon	Pretherapy evaluation	One-to-one training (60 min)	One-to-one training (60 min)	One-to-one training (60 min)	Rest day	One-to-one training (60 min)	Discharge
		Self-exercise (60 min)	Self-exercise (60 min)	Self-exercise (60 min)		Self-exercise (60 min)	
